# Compositional biases in RNA viruses: Causes, consequences and applications

**DOI:** 10.1002/wrna.1679

**Published:** 2021-06-21

**Authors:** Eleanor R. Gaunt, Paul Digard

**Affiliations:** ^1^ Department of Infection and Immunity The Roslin Institute, The University of Edinburgh Edinburgh UK

**Keywords:** dinucleotides, mutation bias, selection bias, viral genome composition

## Abstract

If each of the four nucleotides were represented equally in the genomes of viruses and the hosts they infect, each base would occur at a frequency of 25%. However, this is not observed in nature. Similarly, the order of nucleotides is not random (e.g., in the human genome, guanine follows cytosine at a frequency of ~0.0125, or a quarter the number of times predicted by random representation). Codon usage and codon order are also nonrandom. Furthermore, nucleotide and codon biases vary between species. Such biases have various drivers, including cellular proteins that recognize specific patterns in nucleic acids, that once triggered, induce mutations or invoke intrinsic or innate immune responses. In this review we examine the types of compositional biases identified in viral genomes and current understanding of the evolutionary mechanisms underpinning these trends. Finally, we consider the potential for large scale synonymous recoding strategies to engineer RNA virus vaccines, including those with pandemic potential, such as influenza A virus and Severe Acute Respiratory Syndrome Coronavirus Virus 2.

This article is categorized under:

RNA in Disease and Development > RNA in Disease
RNA Evolution and Genomics > Computational Analyses of RNA
RNA Interactions with Proteins and Other Molecules > Protein‐RNA Recognition

## Graphical abstract

Nucleotide sequences in the genomes of viruses and the hosts they infect are highly patterned; this review discusses the causes, consequences and applications of these genomic compositional biases.

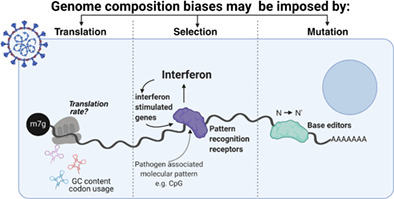

## 1 INTRODUCTION

Synonymous or “silent” nucleotide substitutions in a genome are nucleotide changes that do not result in an amino acid change. The impact of synonymous changes are nevertheless far from phenotypically silent, and have been shown to affect the encoded transcripts in several ways, including mRNA secondary structure (Kudla et al., 2009; Shabalina et al., 2006), mRNA splicing (Warnecke et al., 2009), mRNA stability (Presnyak et al., 2015), microRNA targeting (Birnbaum et al., 2012; Brest et al., 2011), co‐translational protein folding (Pechmann & Frydman, 2013), and, in the context of RNA virus transcripts and genomes, cellular sensing of pathogens (Takata et al., 2017). Synonymous changes are possible due to the phenomenon of codon degeneracy; the potential for one amino acid to be encoded by multiple nucleotide triplets. Codon degeneracy allows viruses a degree of genome plasticity that, by facilitating the evolution of overlapping open reading frames (ORFs) permits the generation of sometimes astonishing genome compression. Nevertheless, while roughly one third of bases in a coding sequence could undergo substitution synonymously, this does not happen, and bases in positions that would theoretically support silent substitution are not randomly represented. In some cases, this is because of superimposed functional elements, such as overlapping ORFs or *cis‐*acting RNA signals. As examples, internal ribosome entry sites (IRESs), first discovered in the *Picornaviridae* (Jang & Wimmer, 1990; Trono et al., 1988), are RNA structures formed over hundreds of nucleotides that enable cap‐independent translation initiation, and silent mutations can impair IRES function. Likewise, orthomyxoviruses package the correct selection of segments through RNA–RNA interactions partly mediated by the coding regions of the genes and again, synonymous mutations can be functionally deleterious (Li et al., 2021). However, synonymous recoding of virus genomes while avoiding known functional elements can nevertheless significantly attenuate virus replication, indicating further constraints on genome sequence acting at a more global level. At this genome level, preferential selection of particular nucleotides at synonymous sites has been previously identified to result in various types of compositional bias, including nucleotide bias (Auewarakul, 2005; Balzarini et al., 2001; Berkhout et al., 2002; Grantham et al., 1980; Jenkins et al., 2001; Kapoor et al., 2010; Lobo et al., 2009; Müller & Bonhoeffer, 2005; Rothberg & Wimmer, 1981; Shackelton et al., 2006; van der Kuyl & Berkhout, 2012; van Hemert et al., 2007; van Hemert & Berkhout, 2016), codon usage bias (Adams & Antoniw, 2004; Aragonès et al., 2010; Bahir et al., 2009; Belalov & Lukashev, 2013; Berkhout et al., 2002; Bouquet et al., 2012; Butt et al., 2014; Cai et al., 2009; Chen, 2013; D'Andrea et al., 2011; Fu, 2010; Grantham et al., 1980; Haas et al., 1996; He et al., 2017; Jenkins et al., 2001; Jenkins & Holmes, 2003; Kumar et al., 2016; Li et al., 2012; Liu et al., 2010; Nougairede et al., 2013; Plotkin & Dushoff, 2003; Rothberg & Wimmer, 1981; Tao et al., 2009; van Hemert et al., 2007; Wong et al., 2010; Zhao et al., 2003; Zhong et al., 2007), dinucleotide bias (Antzin‐Anduetza et al., 2017; Atkinson et al., 2014; Coffin et al., 1995; Di Giallonardo et al., 2017; Gaunt et al., 2016; Karlin et al., 1994; Kunec & Osterrieder, 2016; Rima & McFerran, 1997; Rothberg & Wimmer, 1981; Shackelton et al., 2006; Simmonds et al., 2015; Tao et al., 2009; Tulloch et al., 2014; Upadhyay et al., 2014; Washenberger et al., 2007; Witteveldt et al., 2016) and codon pair bias (Coleman et al., 2008; Gao et al., 2015; Le Nouën et al., 2014; Leifer et al., 2011; Li et al., 2018; Martrus et al., 2013; Mueller et al., 2010; Ni et al., 2014; Wang et al., 2015; Yang et al., 2013). Each of these are elaborated below, along with our current understanding of the underlying mechanisms by which these biases are generated.

While the focus of this review is on genome compositional biases of RNA viruses, often the leading research in a specific area has been undertaking using a DNA virus as a model system, and so where appropriate this research is also described. It is important to note that the concepts discussed have been evaluated using diverse virus systems, often with fundamentally different replication strategies. Exposure to cellular factors is expected to vary depending on where in the cell a virus replicates, the extent of protection of viral genomes from the cellular environment by nucleoproteins, the kinetics of virus replication, as well as the host species and the cell type infected. Nevertheless, all viruses produce mRNAs that are translated in the cytoplasm, so some generalities are likely to exist, as well as differences.

## 2 TYPES OF GENOME COMPOSITIONAL BIAS

### 2.1 Nucleotide bias

If all bases were represented equally in a genome, each would be recorded at a frequency of 25%. However, biases in individual base frequencies are seen across all genomes, including viral. This is often facilitated by codon degeneracy. Of 20 amino acids, 2 are encoded by a unique codon (Met, Trp); nine by two codons (Phe, Tyr, His, Gln, Asn, Lys, Asp, Glu, Cys); Ile is encoded by three codons; five amino acids are encoded by four codons (Val, Pro, Thr, Ala, Gly) and three are encoded by six codons (Leu, Arg, Ser). Representation of each of the degenerate codons can be highly skewed. For example, across the HIV‐1 genome, ~37% of bases are adenine, and adenines are heavily selected for at degenerate positions (Kypr & Mrázek, 1987). This bias is at least partly induced by the cellular factor APOBEC3G (Sheehy et al., 2002), which deaminates cytidine to uridine in the negative sense ssDNA produced during virus replication as an intrinsic antiretroviral defense. Uridine mimics thymine, and so when positive sense DNA is synthesized during genome replication, this reverse complement strand incorporates adenine in place of guanine. This is, in other words, ***mutationally driven***. Conversely, enrichment for adenine at specific sites is thought to reduce the impact of ribosomal frame‐shift events due to introduction of out‐of‐frame stop codons, as modeled using bacterial genomes (Abrahams & Hurst, 2017) (i.e., driven by ***selection***). Other types of nucleotide biases are also described, such as the 70% GC content of the rubella virus genome (Zhou et al., 2012), largely attributed to the use of C bases at degenerate positions(Zhou et al., 2012). Contrarily, extensive C to U mutations (in comparison to other base changes) are seen in the genome of SARS‐CoV‐2 (Rice et al., 2020; Simmonds, 2020). The mechanisms driving these latter two biases are, at present, poorly understood.

### 2.2 Codon usage biases

Usage of degenerate codons is nonrandom, with some codons used frequently and others rarely. Codon preferences vary by host and by viral species, and even by gene. In humans, codon usage biases are stronger in genes that are more highly expressed. The greater exposure of the transcripts from these genes to the drivers of selection may generate stronger biases (Urrutia & Hurst, 2003). Commonly expressed genes use codons which are decoded by abundant tRNAs, whereas during stress the tRNA pool changes to increase abundance of rare tRNAs, as stress response genes are more likely to use rare codons (Torrent et al., 2018). Within‐gene biases are also evident; for example, evolutionarily constrained exonic splice enhancer sites demonstrate different codon usage patterns to other coding regions (Savisaar & Hurst, 2018).

In virology, how well a virus reflects the codon usage of its host can be calculated using the Codon Adaptation Index (CAI) metric. Key to genome composition variation is how long a virus has been adapting to its host; for example, a virus that has recently switched host may change its genome composition profile as it adapts to a new host (Babayan et al., 2018; Greenbaum et al., 2008). In CAI scoring, the most frequently used codons score highly and rare codons score below 1. The scores can then be averaged across an ORF or a proteome. CAI scores vary between −1 and +1, with higher scores representing more frequently used codons with respect to the host (Sharp & Li, 1987). Viral genomes display codon usage biases, but these do not necessarily mimic their host. This may arise as a consequence of nucleotide biases; for example, HIV‐1 and rubella virus display very different codon usage profiles to each other and the human genome as a result of the nucleotide biases they exhibit (van der Kuyl & Berkhout, 2012; Zhou et al., 2012). Genome architecture and virus ecology may also be important for driving codon usage preferences, as codon usage biases may be more evident in segmented and aerosol‐borne viruses compared with vector‐borne viruses (Jenkins & Holmes, 2003), as vector‐borne viruses must also be able to replicate in their invertebrate hosts (Fros et al., 2021). Within viral genomes, codon usage preferences may also vary. For some large DNA viruses, distinct temporal phases of infection occur; usage of rare codons in late genes of large DNA viruses has been proposed as a mechanism of gene expression regulation (Shin et al., 2015; Zhou et al., 1999). In the SARS‐CoV‐2 genome, E ORF and ORF10 encode a high proportion of disfavoured codons, whereas in other genes, codon usage is more reflective of the human host (Digard et al., 2020; Rice et al., 2020).

While the reason(s) underlying codon preferences are somewhat speculative, successive codons encoding the same amino acid are more likely to use the same degenerate base and so the same tRNA, possibly allowing for faster recycling of tRNAs, if tRNA diffusion away from the ribosome happens slower than the rate of translation (Cannarozzi et al., 2010). A nonexclusive alternative is that use of rare codons slows translational rate, which in turn can affect how a protein folds (Kimchi‐Sarfaty et al., 2007).

### 2.3 Dinucleotide biases

**FIGURE 1:**
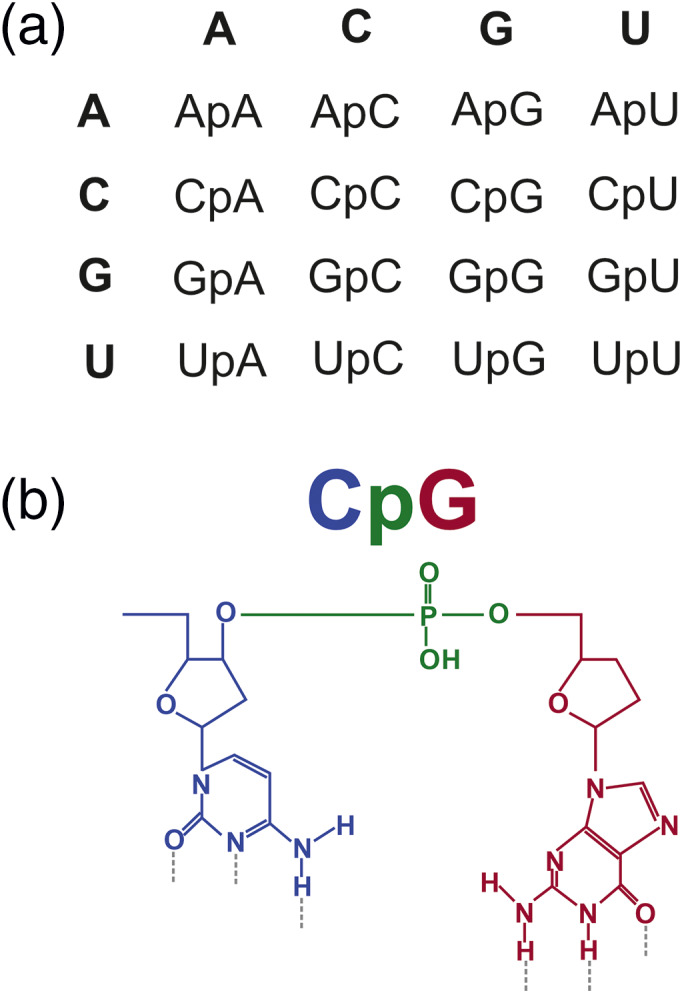
(a) There are 16 possible dinucleotide compositions in RNA. (b) Schematic of CpG motif, with “p” referring to the phosphate bridge (green) joining the cytosine (C) (blue) and guanine (G) (red) bases

In 1981 it was first proposed that nucleotide and codon preferences might be explained by dinucleotide biases (Nussinov, 1981). A dinucleotide is defined as two adjacent nucleotide bases joined by a phosphate bridge, on the same strand of nucleic acid (i.e., in *cis*). Given the four bases of RNA—adenine (A), cytosine (C), guanine (G) and uracil (U)—all possible combinations give rise to 16 possible dinucleotides (Figure 1a). The conventional notation for dinucleotides of, for example “CpG,” refers to a cytosine 5′ to a guanine base and joined by a phosphate (“p”) bridge (Figure 1b).

In a given sequence, how often a given dinucleotide would occur if nucleotide sequence was random can be calculated by simply multiplying observed base frequencies together. By then counting how many times the chosen dinucleotide occurs in a given sequence, over‐ or under‐representation of any dinucleotide can be calculated. This is referred to as the observed: expected (O:E) ratio, represented by the formula:

$$O:E=f\left(\mathrm{XpY}\right)/f\left(X\right).f\left(Y\right)$$

where X and Y represent the two nucleotides of choice. A ratio of above 1 indicates that the observed frequency is higher than expected, and so the dinucleotide is over‐represented, whereas anything below 1 indicates an under‐represented dinucleotide. As an example, consider the CpG O:E ratio for the A/Puerto Rico/8/1934 (PR8) strain of H1N1 influenza A virus (IAV). In PR8, there are 3298 cytosines and 2595 guanines out of a total genome size of 13,588 nucleotides (summed across 8 segments). Thus, the frequencies of C and G are 0.243 and 0.191 respectively. There are 285 CpG motifs out of 13,581 dinucleotides, or an observed CpG ratio of 0.021.

$$\mathrm{CpG}\;O:E\;\text{ratio}\;\text{for the PR8}\;\mathrm{IAV}\;\text{strain}=0.021/\left(0.243\times 0.191\right)=0.453.$$

This simplest method of calculating dinucleotide representation does not take into consideration potential sources of exogenous bias such as amino acid composition and codon bias, although software accounting for such factors has been released (Simmonds, 2012); in our experience of analyzing viral genomes, the results delivered by different models are very similar.

### 2.4 CpG dinucleotides

**FIGURE 2:**
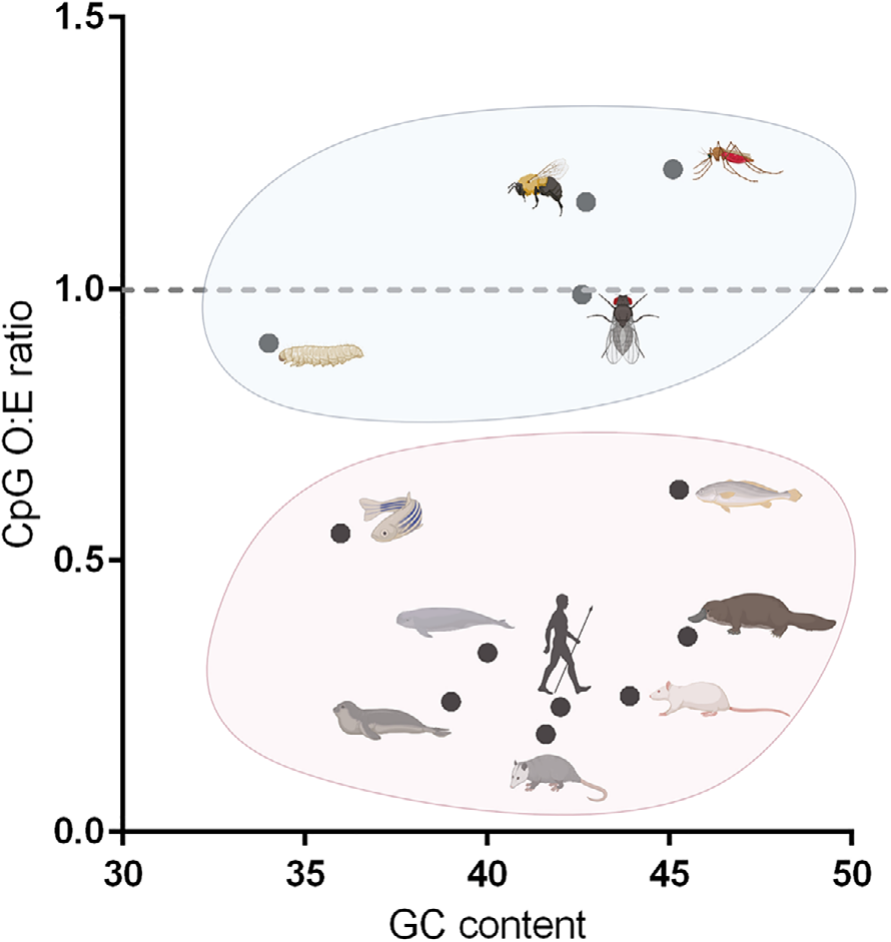
GC content vs CpG ratio for various invertebrate (blue circle) and vertebrate (pink circle) species. In blue from left to right: *Spodoptera exempta* (African armyworm), *Drosophila melanogaster* (fruit fly*), Bombus bombus* (bumble bee), *Anopheles gambiae* (mosquito). In pink from left to right: *Danio rerio* (zebrafish), Halichoerus spp (seals), Phocoena spp (porpoise), *Didelphis virginiana* (opossum), *Homo sapiens* (human), *Rattus norvegicus* (brown rat), *Takifugu rubripes* (pufferfish), *Ornithorhynchus anatinus* (platypus)

Vertebrate genomic dinucleotide composition has been studied since the 1960s, when the striking observation was made that CpG motifs are under‐represented in vertebrate genomes (Swartz et al., 1962; Josse et al., 1961). The human genome has a CpG O:E ratio of around 0.25 (Bird, 1980), similar to other mammalian species (Jabbari et al., 1997) (i.e., CpGs occur at a quarter of the frequency one would expect, given individual cytosine and guanine frequencies in the human genome). Little if any CpG suppression is seen in the genome of invertebrates (Josse et al., 1961; Simmonds et al., 2013) (Figure 2), although CpG suppression is seen in plant genomes (Bougraa & Perrin, 1987; Ibrahim et al., 2019).

In vertebrates, genomic CpG suppression is thought to have arisen due to the epigenetic regulation of transcription occurring in part through the methylation of cytosines in the CpG conformation. Methylated cytosines are prone to undergo spontaneous deamination and so conversion to thymine (i.e., TpG), which is proposed to have resulted in a loss of CpG motifs from vertebrate genomes over evolutionary time (Cooper & Krawczak, 1989). Methylation of cytosines in invertebrate genomes is restricted or entirely absent (Bird & Tweedie, 1995), providing an explanation for the contrasting lack of CpG suppression in these organisms. The reasons for CpG suppression in plant genomes are unclear, as they do not support methylation (Bougraa & Perrin, 1987).

**FIGURE 3:**
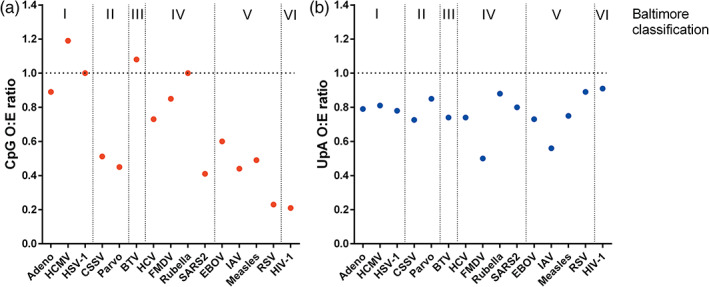
Under‐representation of CpG dinucleotides (a) and UpA dinucleotides (b) in the genomes of representative viruses. Abbreviations are Adeno, human adenovirus 2; HCMV, human cytomegalovirus; HSV‐1, herpes simplex virus 1; parvo, parvovirus; BTV, bluetongue virus; HCV, hepatitis C virus; FMDV, foot and mouth disease virus; SARS2, severe acute respiratory syndrome coronavirus 2; EBOV, ebola virus; IAV, influenza A virus; RSV, respiratory syncytial virus; HIV‐1, human immunodeficiency virus 1. The Baltimore classifications are **I** dsDNA; **II** ssDNA; **III** dsRNA; **IV** +ssRNA; **V** –ssRNA; **VI** rtRNA

In the 1990s it was reported that the genomes of small, but not large, viruses infecting eukaryotes also under‐represent CpG (Karlin et al., 1994). A more detailed analysis (Simmonds et al., 2013) showed that generally in viruses of mammals, single stranded RNA (ssRNA) viruses under‐represent CpG, whereas dsRNA and large DNA viruses do not (Simmonds et al., 2013). The under‐representation of CpG in the IAV PR8 genome described above is therefore characteristic of its class of RNA viruses. By comparison, CpG suppression is less apparent or entirely absent in invertebrate viruses (Simmonds et al., 2013). Viral CpG content can therefore be approximated using the genome type‐based Baltimore classification of viruses (Baltimore, 1971) except in the case of dsDNA viruses, where size matters (Simmonds et al., 2013). Viruses under‐representing CpG in their genomes include the groups of +ssRNA, −ssRNA, small dsDNA, ssDNA (which generally have small genome sizes), positive sense ssRNA reverse transcriptase viruses, and dsDNA reverse transcriptase viruses, while those that do not are dsRNA and large dsDNA viruses (Figure 3a). Overall, for RNA viruses, the extent of CpG bias is considered to be reflective of host (Simmonds et al., 2013). The mechanistic underpinnings giving way to varied rates of CpG suppression are likely to vary between, and even within, different Baltimore group virus classifications due to the differing cellular environments each type of viral genome is exposed to, as well as the different ways in which viruses regulate the cellular environment.

### 2.5 UpA dinucleotides

Dramatic dinucleotide suppression in the genome of vertebrates is unique to CpG. However, the TpA dinucleotide is modestly under‐represented in the genomes of vertebrates, invertebrates (Simmonds et al., 2013) and plants (Bougraa & Perrin, 1987). Both RNA and DNA viruses mimic their host by displaying moderate suppression of the UpA dinucleotide (Di Giallonardo et al., 2017), but to varying extents (Figure 3b).

### 2.6 Codon pair bias

During translation, a ribosome decodes two codons simultaneously, and so as well as codon usage, codon order is also important. Some codon pairs are used more frequently than others, and this is considered as a separate phenomenon of “codon pair bias.” Codon pairs may occur at different frequencies to those expected given the individual codon frequencies within a proteome (Buchan & Stansfield, 2005; Irwin et al., 1995) and in many organisms, some codon pairs are heavily underused, or “disfavoured.” The phenomenon was first described in 1985 in *Escherichia coli* (Yarus & Folley, 1985) and has since been summarized for three domains of life (bacteria, archaea, and eukaryotes) (Tats et al., 2008) (Table 1).

**TABLE 1:** Most strongly avoided codon pairs across bacteria, archaea and eukaryotes

Codon pair	% of organisms which avoid it	O:E ratio
UUC GCA	86	0.570
GGG GGU	83	0.460
UUC GAA	82	0.590
CUU AUG	79	0.529
GCU AUG	76	0.590
ACU AUG	73	0.611
GUU AGC	73	0.529
CUU AGU	73	0.521
UUC GCG	72	0.559
GUU AUG	72	0.611

*Source*: Adapted from Tats et al. (2008).

Codon pair biases impact translation elongation rate (Gamble et al., 2016). In bacteria, over‐represented codon pairs are translated more slowly than under‐represented codon pairs (Irwin, Heck, and Hatfield 1995). Conversely, in eukaryotic cells, 17 specific codon pairs impede translation (Table 2), and reversing their order abrogates the effect (Gamble et al., 2016). These 17 codon pairs were all associated with wobble decoding interactions—that is, a non‐Watson–Crick interactions between the third base of the codon and the first base of the tRNA anticodon. None of these codon pairs are common to those listed in Table 1.

**TABLE 2:** Codon pairs which are inefficiently translated and associated with wobble decoding

Codon pair	First codon wobble	Second codon wobble
AGG CGA	—	I**∙**A
AGG CGG	—	—
AUA CGA	—	I**∙**A
AUA CGG	—	—
CGA AUA	I**∙**A	—
CGA CCG	I**∙**A	U**∙**G
CGA CGA	I**∙**A	I**∙**A
CGA CGG	I**∙**A	—
CGA CUG	I**∙**A	U**∙**G
CGA GCG	I**∙**A	U**∙**G
CUC CCG	—	U**∙**G
CUG AUA	U**∙**G	
CUG CCG	U**∙**G	U**∙**G
CUG CGA	U**∙**G	I**∙**A
GUA CCG	—	U**∙**G
GUA CGA	—	I**∙**A
GUG CGA	—	I**∙**A

*Note*: I**∙**A, inosine base pairing with adenine; U**∙**G, uracil base pairing with guanine.

*Source*: Adapted from Gamble et al. (2016).

Codon pair biases have also been linked with determining efficiency of protein folding and the co‐ordinated expression of functionally grouped proteins (reviewed in Novoa & Ribas de Pouplana, 2012).

The first study of codon pair bias deoptimization of a virus genome determined that in poliovirus, artificially introduced rare codon pairs (relative to host) were translated more slowly (Coleman et al., 2008); this finding has been recapitulated in other virus systems including Marek's disease herpesvirus (Eschke et al., 2018) and IAV (Groenke et al., 2020).

**FIGURE 4:**
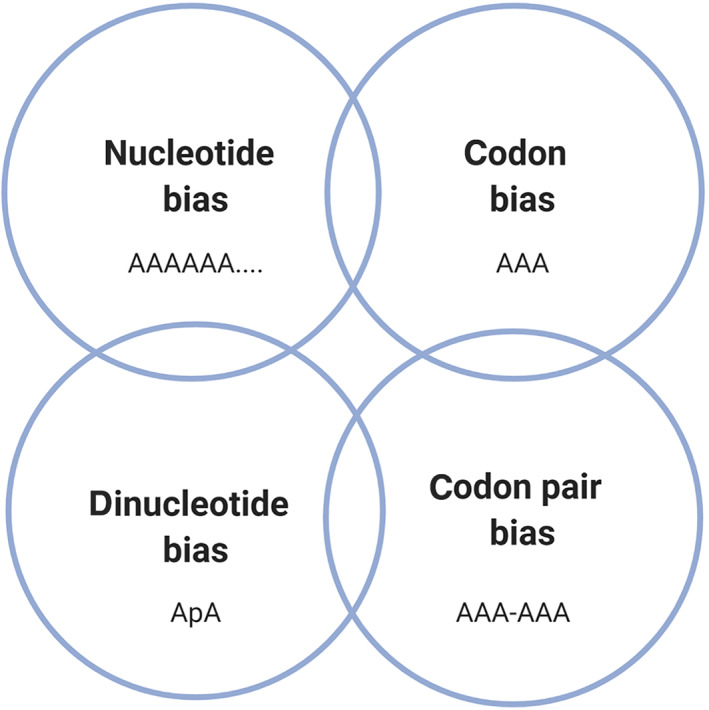
Four types of bias are described in the genomes of organisms and the viruses they are infected with

We have described four different types of bias observed in genomes of organisms and the viruses that infect them—nucleotide bias, codon bias, dinucleotide bias and codon pair bias. Let us reconsider the HIV‐1 genome—the A base is highly over‐represented, occurring with a frequency of ~37% (Kypr & Mrázek, 1987). If we did not know the underlying mechanism causing this bias, we may have difficulty determining which type of bias we were looking at, because all four may look similar (Figure 4). In order to deconvolute these types of bias, we need to understand the underlying mechanisms underlying their presence in more detail.

## 3 DRIVERS OF VIRAL GENOME COMPOSITIONAL BIAS

**FIGURE 5:**
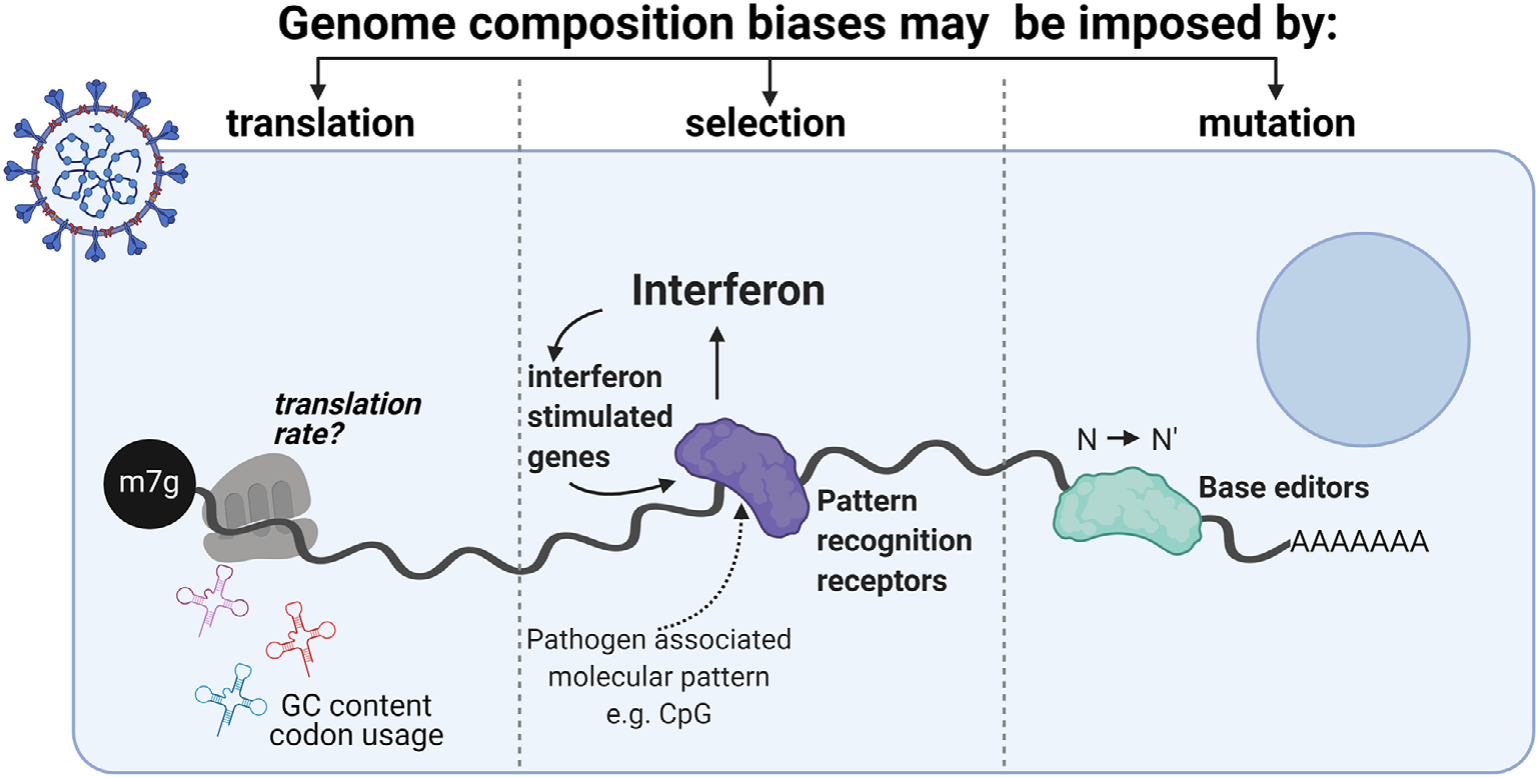
Compositional biases in viral genomes may be driven by three types of evolutionary pressure—Translational, selection and mutational. Translationally derived biases arise due to the different translational efficiencies of transcripts with varying composition in different cell conditions (e.g., resting vs. stress). Biases driven by selection arise through viral genomes avoiding encoding specific motifs that may be recognized by components of the innate immune response. Biases driven by mutation arise through editing of viral genomes or transcripts by host cell proteins

As described above, genomic composition biases may arise through a variety of evolutionary selection pressures, both positive and negative. These potential drivers of bias are summarized below and in Figure 5.

### 3.1 Biases driven by factors influencing translational rate

The efficiency with which different codons and codon pairs are translated in resting cells compared with stressed cells (e.g., during virus infection) varies depending on the tRNA pool available (Buchan et al., 2006). In a study that examined translational efficiency of a library of 217 synonymously recoded GFP sequences, codon usage and GC content of genes were both found to influence translational efficiency, mRNA splicing efficiency and mRNA subcellular localization (Mordstein et al., 2020). In addition, in resting cells, high GC content of a gene increases its transcriptional rate (Kudla et al., 2006). Whether these features influence the translational efficiency of viral genes, and whether viral genes have evolved specific composition traits to regulate transcription and translation, is unknown, but the hypotheses are reasonable. Use of codons or codon pairs which require wobble decoding is known to increase the likelihood of mistranslation events (Patil et al., 2012), and mistranslation events are more frequent during cellular stress (Mohler & Ibba, 2017). Wobble decoding contributes to increased access to alternative reading frames (Drummond & Wilke, 2009; Ou et al., 2019), and so may be relevant for viruses which encode overlapping reading frames, but whether these events are physiologically important for viral replication is also unknown. Translational fidelity can nevertheless shape virus evolution (Ou et al., 2019); for example, some mitochondrially replicating mitoviruses avoid use of tryptophan codons, which mirrors avoidance of their use by the host fungi organelle mitochondrial genome (Nibert, 2017). RNA modifications (e.g., m^6^A methylation) may also regulate translation (reviewed elsewhere; Roundtree et al., 2017) and the frequency of such modifications is related to biases in individual base frequencies.

### 3.2 Biases driven by factors influencing mutation

Mutations arise in viral genomes either through the actions of host cell editors (i.e., direct *mutation)*, or by copying errors that then become fixed in the viral genome (*selection*). We have already considered the A‐rich genome of HIV‐1, and understand that this has arisen due to the *mutational* activities of the cellular protein APOBEC3G. Similarly, the cellular proteins of the adenosine deaminase acting on RNA (ADAR) family convert adenosine to inosine; evidence for ADAR acting on virally derived nucleic acids was first reported in the genome of vesicular stomatitis virus (O'Hara et al., 1984) but has since been identified in the genomes of a range of other viruses (Samuel, 2012). There are numerous other APOBEC and ADAR family members with potential to act on viral genomes (Christofi & Zaravinos, 2019). The observation that the SARS‐CoV‐2 genome is extremely uracil‐rich (Rice et al., 2020; Simmonds, 2020) has been speculatively attributed to the editing efforts of cellular mutators such as APOBEC (originally reported to edit DNA, but also reported to act on RNA; Sharma et al., 2016) and ADAR (Simmonds, 2020; Di Giorgio et al., 2020), but could also be attributable to an as‐yet unidentified cellular protein.

### 3.3 Biases driven by factors influencing selection

*Selection* pressure might arise also due to the activities of a cellular protein that, for example, recognizes a specific viral motif or pathogen‐associated molecular pattern (PAMP). In general, recognition of a viral PAMP by a host cell protein (or a “pattern recognition receptor”; PRR) triggers type I interferon signaling; these PRRs may themselves be upregulated by interferon, and in this case are known as interferon stimulated genes (ISGs) (reviewed in [Kumar et al., 2011]). The concept of PAMPs being recognized by PRRs during the innate immune response was first hypothesized by Charles Janeway in 1989 (Janeway, 1989). As he predicted, the first PRR identified was Xa21, a gene that protects rice from bacterial infection (described in 1995) (Song et al., 1995). Of the many current examples of PRRs, some recognize specific viral nucleic acid signatures and thus may contribute to driving genome compositional biases. The 10 Toll‐like receptors (TLRs) identified in humans are heavily evolutionarily conserved across vertebrates (Oshiumi et al., 2008) and some can recognize pathogen nucleic acids. The clearest example of this relevant to compositional biases is that TLR9 recognizes unmethylated CpG motifs in DNA (Bauer et al., 2001; Krug et al., 2004; Tabeta et al., 2004), and genomic suppression of CpG in murine herpesvirus 68 to evade detection by TLR9 has been reported (Pezda et al., 2011). Examples for RNA viruses are less clear‐cut, but TLR7 recognizes purine‐rich viral ssRNA (Gantier et al., 2008; Zhang et al., 2016). Thus, deselection of these PAMPs over evolutionary time may be due to the selection pressures applied by these PRRs, as well as as‐yet‐unidentified cellular factors.

### 3.4 Mechanistic understanding of how viral CpGs are selected against

The suppression of CpG dinucleotides in the genomes of viruses and their hosts illustrates a fascinating contrast between mutational versus selection pressure. As described above, over evolutionary time the deamination of methylated CpG motifs in vertebrate genomes has resulted in their removal by mutation (biases driven by mutation). Viral mimicry of genomic CpG suppression was hypothesized to be due to aberrant CpG frequency sensing by an as yet unidentified PRR (Atkinson et al., 2014), and thus CpG motifs had been deselected in viral genomes (biases driven by selection). This hypothesis was strengthened in 2017, when a breakthrough paper reported that the product of the cellular ISG, zinc‐finger antiviral protein (ZAP) senses CpG motifs in viral RNA (Takata et al., 2017). ZAP has long been identified as a suppressor of some but not all viruses by inducing degradation of specific viral mRNAs through an unknown targeting mechanism (Gao et al., 2002; Guo et al., 2007; Bick et al., 2003; Zhu et al., 2011). This more recent study used the HIV‐1 genome as a model system in which to synonymously enrich CpG frequencies, and while the mutant virus was replication defective in normal cells, that defect was fully abrogated in a ZAP knockout system (Takata et al., 2017). Similarly, enrichment of CpGs in the echovirus 7 genome also caused a replication defect, that could be restored by ZAP knockout (Odon et al., 2019). Similarly, an inhibitory role for ZAP against human cytomegalovirus has been shown, which correlated with CpG‐content dependent inhibition of viral Immediate Early 1 protein expression (Lin et al., 2020), further strengthening evidence that ZAP acts as an antiviral PRR though sensing high CpG frequencies in viral mRNAs.

ZAP is encoded on the ZC3HAV1 gene, which generates multiple isoforms via alternative splicing. Two isoforms are expressed to levels readily detectable by western blotting: the long (ZAPL) and short (ZAPS) forms (Li et al., 2019). From the N terminus, both major isoforms incorporate four zinc fingers implicated in RNA binding (Guo et al., 2004), a TiPARP Homology (TPH) domain, also containing a zinc finger (Kerns et al., 2008), and a WWE domain predicted to mediate interactions with proteins that facilitate post‐translational conjugations (Aravind, 2001). In comparison with ZAPL, ZAPS lacks the catalytically inactive poly(ADP‐ribose) polymerase (PARP)—like domain, which enhances antiviral activity against an alphavirus and a retrovirus (Kerns et al., 2008). ZAPL is considered to be the constitutively expressed isoform, whereas ZAPS is an ISG which itself triggers IFN (Hayakawa et al., 2011; Ryman et al., 2005; Marcello et al., 2006) and is implicated in CpG recognition (Takata et al., 2017). Accordingly, here we only consider ZAPS (and refer to it simply as “ZAP”). The original paper reporting ZAP as a CpG sensor demonstrated the specific binding of ZAP at CpG sites using cross‐linking followed by immunoprecipitation (CLIP) and sequencing (Takata et al., 2017). Crystallographic resolution of the structure of the N‐terminus of ZAP bound to CpG motif‐containing RNA revealed that the four zinc fingers of ZAP fold in a specific architecture to enable extensive RNA interactions which were diminished by mutation either of RNA CpG sites, or of ZAP at the zinc finger motifs (Luo et al., 2020; Meagher et al., 2019).

**FIGURE 6:**
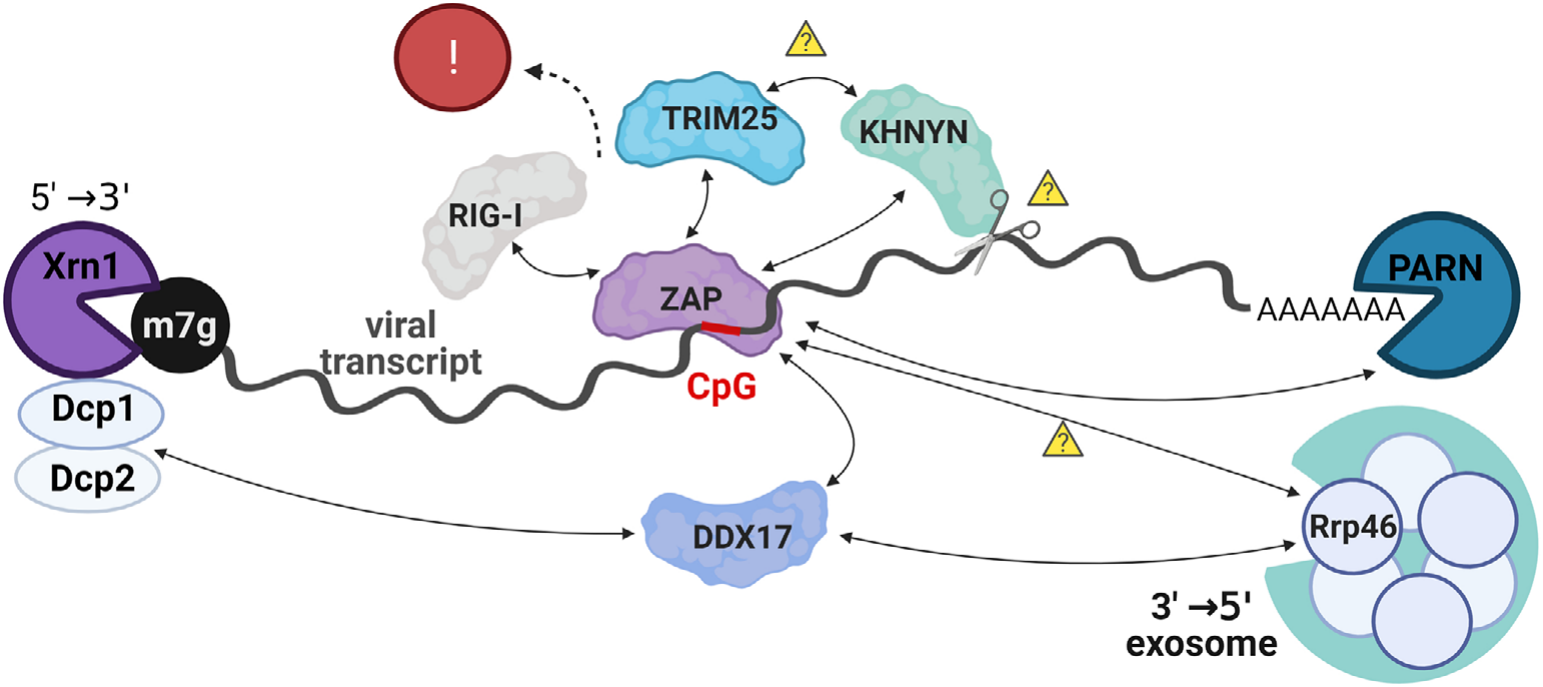
Possible mechanisms by which ZAP activity leads to viral transcript degradation. CpG motifs in viral RNA (red) are bound by the cytoplasmic PRR ZAP, which can lead to recruitment of 5′ decapping enzymes (Dcp1/2 complex), the 3′ deadenylation enzyme PARN and potentially the KHNYN RNA endonuclease, followed by 5′–3′ degradation mediated by Xrn1 and/or 3′–5′ degradation mediated by the RNA exosome. Interactions between ZAP and RIG‐I and/or TRIM25 may also lead to innate immune signaling

Following ZAP recognition of CpG‐containing RNA, antiviral activity arises by inhibition of virus gene expression, either by mRNA degradation and/or inhibition of translation (Guo et al., 2007; Zhu et al., 2011). ZAP may inhibit translation by disrupting interactions between the translation initiation factors eIF4A and eIF4G (Zhu et al., 2012). ZAP also recruits transcripts to stress granules (Law et al., 2019). Degradation of viral mRNA is thought to occur through multiple routes, including via recruitment of the RNA exosome complex and/or the major cytoplasmic exoribonuclease, Xrn1 (Guo et al., 2007; Goodier et al., 2015; Todorova et al., 2014; Zhu et al., 2011). ZAP directly interacts with several exosome components, and their depletion by siRNA knockdown resulted in diminished antiviral activity by ZAP (Guo et al., 2007), confirming an essential role for the exosome in ZAP‐mediated RNA degradation. During exosome‐mediated RNA degradation, mRNAs must be deadenylated and then decapped to yield a monophosphorylated RNA, which can then also be digested by Xrn1 (Chang et al., 2019). Interactions between ZAP and poly‐A specific ribonuclease (PARN) may direct deadenylation of the mRNA, while interactions between Xrn1 and the decapping enzymes necessary for 5′ → 3′ RNA degradation are indirect, via the RNA helicase DDX17(Zhu et al., 2011). Xrn1 also digests endonucleolytically cleaved RNAs (Gatfield & Izaurralde, 2004), but it is not definitively known whether ZAP binding leads to internal mRNA cleavage events. In support of this possibility, ZAP binds to and its inhibitory activity against CpG‐enriched transcripts is dependent on the cellular protein KHNYN, which unlike ZAP, does possess endonuclease activity (Ficarelli et al., 2020; Ficarelli et al., 2019). This is summarized (Figure 6).

How ZAP feeds back into the interferon pathway is uncertain. ZAP has been shown to interact with the cytoplasmic PRR RIG‐I and to augment innate immune signaling in response to a variety of artificial RNA stimuli (Hayakawa et al., 2011). This study was performed prior to ZAP's identification as a CpG sensor however, and focussed on recognition of 3′‐triphosphate RNA moieties; it remains to be determined if CpG‐rich RNA signals through the same mechanisms.

Alternatively, ZAP‐mediated innate immune responses may themselves be mediated through interactions with ZAP's cofactor TRIM25. ZAP is directly bound by TRIM25, itself an RNA binding protein and also an E3 ubiquitin ligase (Zou & Zhang, 2006), and this interaction is required for ZAP's antiviral activity (Li et al., 2017; Zheng et al., 2017). TRIM25 binds ZAP through TRIM25's SPRY domain (a protein interaction module characterized by a sequence repeat; D'Cruz et al., 2013) and ubiquitinates ZAP, although ubiquitination is not required for ZAP antiviral activity (Choudhury et al., 2017). TRIM25 was originally understood to be essential for activation of the RIG‐I‐dependent pathway for interferon activation (Gack et al., 2007), but recently it was shown that RIPLET and not TRIM25 ubiquitinates RIG‐I, and that RIPLET is sufficient for the ubiquitination and activation of RIG‐I (Cadena et al., 2019). It is therefore unclear how important TRIM25 (or by extension, the interaction between ZAP and TRIM25) is during virally induced activation of the interferon response.

CpG suppression may be more nuanced than the blanket genome‐wide suppression described above, which has consequent implications for the mechanisms of, and viral counteractivity to, ZAP. In the genomes of Betaherpesviruses, immediate early genes suppress CpG, whereas this is not seen in the rest of the genome (Lin et al., 2020). The authors hypothesized that immediate early gene product(s) are able to abrogate ZAP activity, thus removing any selection against high CpG frequencies in viral genes that are activated at later timepoints during infection. Conversely, in the SARS‐CoV‐2 genome, CpG is over‐represented in E (envelope) ORF and in ORF10, whereas other genes—as expected—suppress CpG (Digard et al., 2020; Rice et al., 2020). Why these ORFs are able to buck the trend seemingly imposed on the rest of the genome is unknown; possibly, high CpG frequencies invite turnover by ZAP, thereby regulating protein production. Alternatively, these ORFs may have been acquired through recombination events and had an ancestral origin not previously subject to the same translational, mutational or selection pressures.

### 3.5 CpG context may be an important driver of biases imparted by selection

For ZAP to function as an innate immune sensor and/or effector for foreign RNAs containing high CpG content, there must be a mechanism to limit activation of the system by cellular RNAs that also contain CpG dinucleotides (as all do). Since ZAP recognizes CpG motifs in ssRNA, it is possible that secondary structure of RNA—i.e., CpG context, is an important factor in determining whether CpG motifs can be recognized by ZAP, and there is some evidence indicating this. First, in the crystallography paper characterizing ZAP‐RNA binding, the optimal binding motif for ZAP on RNA was found to be C(n_7_)G(n)CG (Luo et al., 2020). ZAP was found to bind to multiple sites on an RNA, and in considering the stoichiometry of RNA degradation complex recruitment, the authors concluded that owing to the relatively small size of ZAP relative to RNA degradation complexes, several bound ZAP molecules must be required for this. Therefore the number and spacing of CpG dinucleotides is likely to be important.

Context effects for CpG deselection have also been identified in an evolutionary context. Greenbaum et al., found that since the emergence of the 1918 H1N1 pandemic strain of IAV in humans, CpG motifs have gradually been lost from the viral genome as it became endemic in humans. They asked whether specific nucleotides were more likely to flank the CpGs that were deselected, by measuring the relative frequencies of (C/G)CG(C/G), (A/U)CG(A/U), (A/U)CG(C/G) and (C/G)CG(A/U) in H1N1 genomes over time (Greenbaum et al., 2009). No reduction in (C/G)CG(C/G) motifs was seen, whereas all three of the other motifs declined in frequency, with the strongest reduction seen in the (A/U)CG(A/U) motif. The authors speculated that the severe disease attributed to infection with the 1918 virus was caused by the aberrantly high CpG frequency present in the viral genome provoking a cytokine storm.

A similar observation has been recapitulated in vitro. Using echovirus 7 as a model system, a replicon was recoded to maintain CpG frequency (*n* = 51) but add AACGAA or UUCGUU motifs (Fros et al., 2017). The UUCGUU mutant was fivefold more impaired than a CpG enriched transcript (Fros et al., 2017). Thus, there is a growing body of evidence that CpG context is important for innate sensing.

### 3.6 UpA dinucleotide sensing as a driver of bias

**FIGURE 7:**
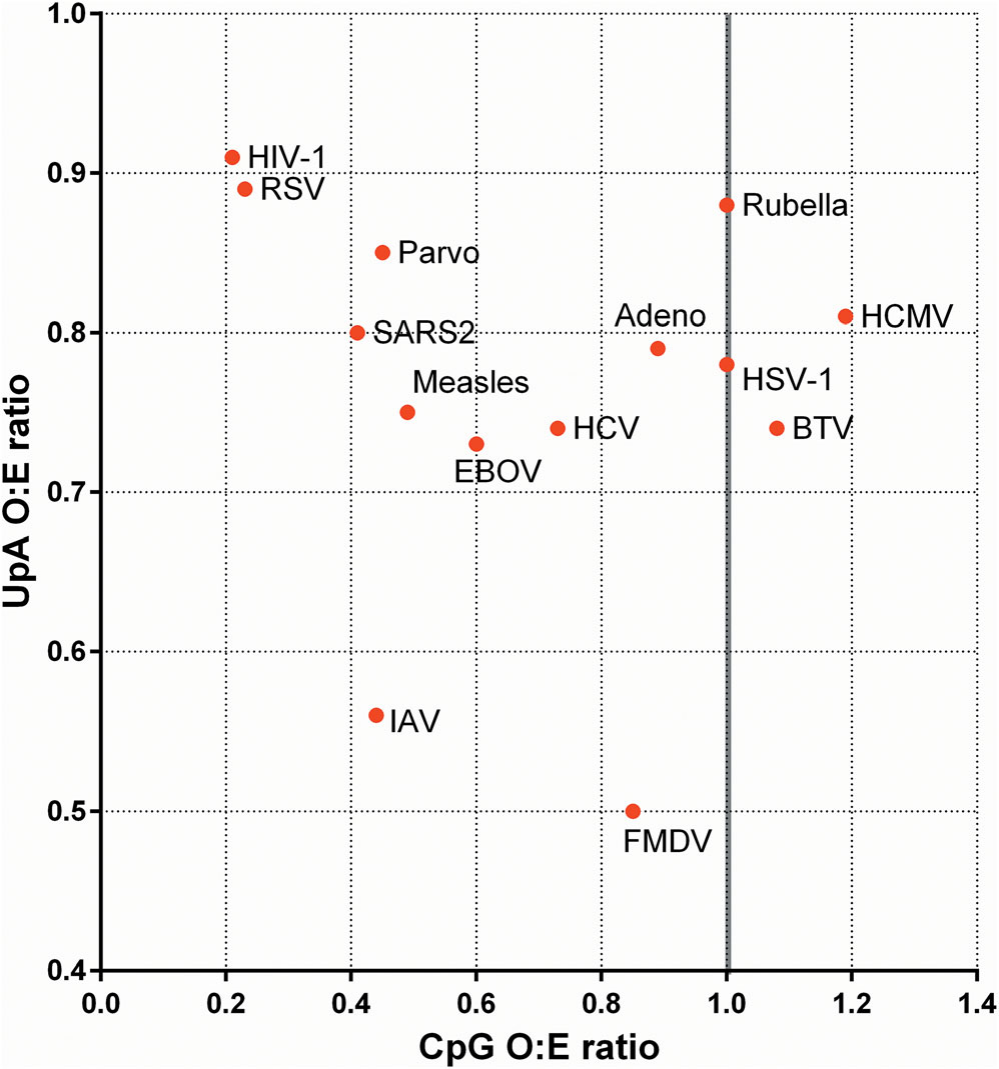
Comparison of CpG and UpA suppression in the genomes of various viruses. RNA viruses: BTV, bluetongue virus; EBOV, ebola virus; FMDV, foot and mouth disease virus; HCV, hepatitis C virus; RSV, respiratory syncytial virus; SARS2, severe acute respiratory syndrome coronavirus 2. DNA viruses: adeno, adenovirus; HCMV, human cytomegalovirus; HSV‐1, herpes simplex virus 1; Parvo, canine parvovirus 2

Two possible explanations have been put forward to date to explain genomic UpA suppression. First, it was originally reported in 1981 (and subsequently verified) that UpA dinucleotides are cleaved by the cellular ISG RNaseL (Wreschner et al., 1981; Karasik et al., 2021), which could explain their deselection over evolutionary time. However, the authors further reported that RNaseL also cleaves RNA at UpU dinucleotides, and TpT/ UpU are generally not under‐represented in animal genomes or in the viruses that infect them, so the specificity and impact of RNaseL on genomic TpA/UpA content is questionable. So far, one study using echovirus 7 as a model found that the reduced replication of an artificially UpA‐enriched virus could be rescued by RNaseL removal (Odon et al., 2019), but it appears that the pathway is not specific to RNaseL, as ZAP depletion also complemented the defect in virus replication. While both CpG and UpA dinucleotide suppression may be driven by co‐regulated factors of the interferon response, the extent of CpG and UpA suppression within a virus genome do not necessarily correlate (Figure 7).

The second nonexclusive idea to explain TpA/ UpA suppression is the propensity for this dinucleotide to introduce a stop codon. Stop codons are encoded by UAG, UAA and UGA nucleotide triplets, and so deselection of UpA motifs in the first and second codon positions reduces the risk of aberrant stop codon introduction. However, 6 of 10 disfavoured codon pairs encode a UpA motif cross the codon boundary (Table 1), and so deselection of UpA motifs in this context is evident and may therefore be important for translation regulation. Therefore, multiple constraints may be acting which, together, reduce UpA representation in the genomes of organisms and their infecting viruses.

## 4 CONSEQUENCES OF ALTERING VIRAL GENOME COMPOSITIONAL BIASES

To study the biological relevance of under‐represented nucleotides, dinucleotides, codons and codon pairs, synonymous recoding has been undertaken for a wide range of viruses. These are summarized (Table 3). In these studies, deoptimization to alter sequence composition in a direction away from that of the host, or optimization to recode viral sequence to look more like host genome has been undertaken. Generally, deoptimization of any of these parameters results in virus attenuation, whereas optimization usually does not improve replication.

**TABLE 3:** Synonymous recoding strategies which have been applied to RNA viruses are summarized

Virus	Recoding strategy	Region recoded	Findings	References
Adeno‐associated virus	Codon pair bias deoptimization	Rep	The negative regulatory signal imparted on adenovirus by AAV was diminished, and so adenovirus replication was enhanced	Sitaraman et al. (2011)
Dengue virus	Codon pair bias deoptimization to match insect bias	E/NS3/NS5	Mutants grow well in insect cells but not well (if at all) in mammalian cells. LD50 was 10^2–3.5^ fold up in mice	Shen et al. (2015)
	Bioinformatic analyses showed that the above recoding strategy also increased CpG frequency		This re‐analysis suggested that attenuation of viral replication in mammalian cells might result from increased CpG content rather than increased codon‐pair bias	Simmonds et al. (2015)
Echovirus 7	CpG or UpA dinucleotide bias optimization and deoptimization	VP3/1 and/or 3B/C/D	CpG enrichment in two regions caused a 7000‐fold reduction in replication; UpA enrichment caused a 30‐fold reduction in cells. Removal of CpGs and UpAs increased replication, with removal of both increasing virus titres 10‐fold in cells	Atkinson et al. (2014)
Foot and mouth disease virus	Codon pair bias deoptimization	P1 capsid	10^3^‐fold increase in the vaccine safety margin compared with WT virus	Diaz‐San Segundo et al. (2016)
Human cytomegalovirus	CpG dinucleotide deoptimization	IE1	Reporter constructs with elevated CpG content triggered ZAP induction	Lin et al. (2020)
Human immunodeficiency virus 1	Codon pair optimization and deoptimization	Gag and pol	No observed effects of optimization; deoptimization reduced replication titre in cells. Deoptimized but not optimized virus reverted following passage	Martrus et al. (2013)
	Increased CpG frequency	Gag	Up to 10^2^‐fold defect in replication in cells	Antzin‐Anduetza et al. (2017)
Influenza A virus	Codon pair bias deoptimization	PB1, NP and HA	10^1^‐fold reduction in titre in cells	Mueller et al. (2010)
	Codon pair bias deoptimization	HA and NA	10^5^‐fold attenuation in mice and clinical attenuation in ferrets	Yang et al. (2013) and Broadbent et al. (2016)
	CpG and UpA dinucleotide deoptimization	NP	10^1–2^‐fold reduction in titre in cell culture and disease attenuation in mice	Gaunt et al. (2016)
	CpG and codon pair bias deoptimization	NA	Codon pair bias dramatically decreased replication whereas increased CpG dinucleotides did not	Groenke et al. (2020)
Poliovirus	Codon usage bias deoptimization	Capsid	65 fold reduction in virus titre in cells	Burns et al. (2006)
	CpG and UpA dinucleotide deoptimization	Capsid	Up to a 10^3^‐fold reduction in virus titre in cells	Burns et al. (2009)
	Codon usage optimization and deoptimization	Capsid	Little effect with codon optimization; deoptimization reduced virus titre in cells and mice	Lauring et al. (2012)
	Codon pair bias deoptimization	Capsid	Replication defect correlated with extent of mutagenesis in cells	Coleman et al. (2008)
Porcine reproductive and respiratory syndrome virus	Codon pair bias deoptimization	GP5	A 10‐fold replication defect in cells, 10^3^‐fold decrease in virus titre in pigs	Ni et al. (2014)
	Codon pair bias deoptimization	NSP9	10^4^‐fold replication defect in cells, no evidence of infection in pigs	Gao et al. (2015)
Potato virus Y	CpG and UpA dinucleotide deoptimization	Nonstructural genes	Up to 10^3^‐fold defect (CpG) or 10^6^‐fold defect (UpA) in systemic spread	Ibrahim et al. (2019)
Respiratory syncytial virus	Codon pair deoptimization	Various combinations, with the most extensive recoding extending to all ORFs except M1 and M2	Multiple log_10_‐fold reduction in titre of various mutants in cells, mice and African Green Monkeys	Le Nouën et al. (2014)
	Codon deoptimization by altering codon usage to be consistent with human	NS1 and NS2	Modest replication attenuation in cells and mice	Meng et al. (2014)
Simian immunodeficiency virus	Nucleotide optimization towards nucleotide frequencies in macaque	Gag and pol	10^2^‐fold decrease in replication in cells; recoding in polymerase only had no effect	Vabret et al. (2014)
Vesicular stomatitis virus	Codon pair bias optimization and deoptimization	Polymerase	Optimization resulted in a modest replication defect in cells and 10^2–3^‐fold deficit in mice. Deoptimized virus could not be recovered	Wang et al. (2015)

### 4.1 Codon pair bias recoding

The first study (published in 2008) to draw significant attention to the subject of large scale genome recoding examined the effects of modifying codon pair bias in the poliovirus genome (Coleman et al., 2008), where deoptimization of codon pairs resulted in virus attenuation, and the extent of recoding correlated with the extent of attenuation. The authors found that introduction of disfavoured codon pairs decreased protein translation rates (assayed using a luciferase reporter construct) and yielded viruses that were attenuated in mice, but still offered protection from homologous virus challenge. Following on from this work, several papers (by the same research group and others) expanded this concept by applying the same recoding strategy to other viruses (Table 3).

The potential for codon pair bias recoding as a vaccine development strategy has also been demonstrated in work using IAV as a model system. The PR8 strain of H1N1 IAV (the backbone of which is used to make live attenuated IAV vaccines) was recoded to increase disfavoured codon pair usage. Three viral genome segments (2 [PB1], 4 [HA], and 5 [NP]) were modified and tested separately or in combination for their effects on viral growth characteristics in cells and vaccine potential in mice. Single or combinatorial segment modifications all displayed around 10‐fold defect in multicycle replication assays in vitro. However, in BALB/c mice, the triple reassortant had a 3000‐fold reduction in virus titre at 24 h post‐infection. The triple reassortant virus was further tested for its 50% protective dose (PD_50_; i.e., the inoculum dose required to protect from infection upon challenge), displaying a 50% lethal dose (LD_50)_)/PD_50_ ratio 1000‐fold higher than that of wildtype PR8 virus. This result, and others from the same lab (Yang et al., 2013; Broadbent et al., 2016) emphasized the potential of large scale genome recoding as an approach to live‐attenuated vaccine development.

A question often posited when large scale recoding of viruses to either mimic or deviate from the patterns seen in host genomes is considered, is what happens in the case of vector‐borne viruses that replicate in both invertebrate and vertebrate hosts. This was investigated for dengue virus, which replicates effectively in both the main insect vector *Aedes aegypti*, and in humans (Olson et al., 1996). Recoding of the dengue virus genome to align its codon pair usage in favor with insect genome preferences resulted in a virus that replicated as well as wildtype in insect cells, but experienced a 1–2 log_10_ decrease in replication in some mammalian cells. In mice, this recoding resulted in a 2–3 log_10_ increase in LD_50_. Curiously, the recoded virus replicated normally in BHK‐21 cells.

### 4.2 Dinucleotide recoding

Large scale recoding of virus genomes using dinucleotide deoptimization was first reported in 2006, using poliovirus as a model system (Burns et al., 2006). In this paper, the authors set out to recode poliovirus by deoptimizing codon usage, but observed that in the process, they introduced 207 CpG dinucleotides across the capsid region. In doing this, virus titres were reduced 65‐fold.

The same group went on to specifically study the impact of CpG and UpA enrichment on poliovirus replication. Addition of CpG or UpA were both found to diminish replication, and when both dinucleotide frequencies were simultaneously increased, the effects were found to be synergistic (Burns et al., 2009).

These first papers investigating dinucleotide deoptimization were confounded by lack of corrections for nucleotide and codon usage biases, which as noted above (Figure 4), are inter‐related. More recent works have enriched CpG and UpA dinucleotides without altering nucleotide or codon frequencies. The introduction of CpGs or UpAs into the echovirus 7 genome in a way that controlled for these other variables (Atkinson et al., 2014) found that CpG introduction more strongly reduced virus fitness than UpA introduction. Conversely, using IAV as a model system, UpA introduction was more detrimental to virus replication than CpG addition. This IAV work also demonstrated that a sub‐clinical dose of CpG‐enriched virus protected from challenge with a potentially lethal dose of the wildtype PR8 strain in mice (Gaunt et al., 2016), directly demonstrating the potential of dinucleotide deoptimization as a vaccine development strategy.

CpG and UpA dinucleotide optimization and deoptimization have been characterized in the genomes of various other viruses (Table 3), but the bulk of these studies were undertaken prior to the discovery of ZAP as a CpG sensor. The defect imparted by CpG enrichment has been abrogated by ZAP knockout in echovirus 7 (Odon et al., 2019) and HIV‐1 systems (Ficarelli et al., 2020; Takata et al., 2017), although for echovirus 7 the defect was also relieved by RNaseL knockout, and CpG enrichment in HIV‐1 impacted splicing events. The role of ZAP in CpG sensing of viral RNAs requires further clarification.

### 4.3 Is codon pair bias an artifact of dinucleotide bias?

Of the top 10 most avoided codon pairs across bacteria, archaea and eukaryotes, three contain a CpG motif at the codon boundary and six contain a UpA motif (Table 1). Thus, the two phenomena of codon pair bias and dinucleotide bias are interlinked and at one extreme could simply be two ways of measuring the same effect. This has proven to be a contentious issue (Simmonds et al., 2015; Futcher et al., 2015). Deconvoluting the two has been achieved using the echovirus 7 system to make a panel of mutants which were either codon pair bias deoptimized or dinucleotide bias deoptimized, without altering the other parameter. Using this system, codon pair bias did not impact virus replication kinetics, whereas dinucleotide composition did (Tulloch et al., 2014). This finding was supported by a bioinformatics study from an independent laboratory which reached the same conclusion (Kunec & Osterrieder, 2016). However, when the same authors from this latter study used IAV as a model system to experimentally test their predictions, they found that codon pair bias was far more important than dinucleotide bias, using IAV as a model system (Groenke et al., 2020). In this latter report, the authors found that codon pair deoptimization resulted in diminished mRNA stability (Groenke et al., 2020); however, the codon pair deoptimization resulted in increased UpA dinucleotide frequencies, and as UpA is reported to be cleaved by RNaseL (Wreschner et al., 1981; Karasik et al., 2021), this could also explain the outcome. A bioinformatics study that used nucleotide patterns of viruses to predict host species found the two features to be discrete, but that dinucleotide bias was far more accurate than codon pair bias in identifying viral host species (Babayan et al., 2018).

The field therefore remains divided about whether codon pair and dinucleotide bias are synonyms or are discrete phenomena. The confusion between codon pair bias and dinucleotide bias is compounded by a proportion of the described studies not including control constructs—e.g., re‐ordering codons without altering dinucleotide frequencies (Atkinson et al., 2014; Fros et al., 2017; Gaunt et al., 2016; Ibrahim et al., 2019; Odon et al., 2019). Such controls are imperfect—perhaps, for example, a deoptimized virus has inadvertently introduced a mutation that alters an uncharacterized RNA functional element, whereas the recoding in the control virus did not. Such controls are nevertheless still helpful for strengthening mechanistic conclusions, even if redundant for purely empirical attempts to attenuate a virus. Furthermore, not all studies fully investigate the mechanism of attenuation—for example, few have assessed translation rates (impacted by codon pair biases) or RNA turnover (impacted by dinucleotide biases). Ultimately, better control strategies are needed to deconvolute these two phenomena properly. Distinction—or not—between dinucleotide bias and codon pair bias can be made if we fully understand the mechanism(s) by which these biases attenuate virus propagation.

The discovery of ZAP as a CpG sensor provides the opportunity for researchers to validate CpG enrichment studies. If CpG enrichment results in a defect which can be abrogated with ZAP knockout, the impairment phenotype can sensibly be concluded to be a result of ZAP activity and therefore the introduction of CpGs (rather than an unintended side effect such as introduction of disfavoured codon pairs). Publications on this so far report mixed results (Ficarelli et al., 2020; Odon et al., 2019; Ficarelli et al., 2019) wherein ZAP is not the only sensor whose depletion results in fitness reconstitution, or off‐target effects of CpG enrichment are seen. Nevertheless, ZAP knockout looks like a promising test of whether CpG enrichment is the key to why a CpG‐enriched virus has a replication defect. However, no such “rescue system” exists for codon pair bias studies. Perhaps, if the limitation is in translational efficiency, tRNA supplementation would rescue the system, but we are not aware of any study attempting this.

## 5 DISCUSSION: POTENTIAL FOR DINUCLEOTIDE MODIFICATION AS A VACCINE DEVELOPMENT STRATEGY

The detailed observations on the large‐scale recoding of RNA virus genomes has enthused researchers to repeatedly suggest that these methods may offer a potential live attenuated vaccine development strategy, as described above for both codon pair bias and dinucleotide bias deoptimization.

A critical consideration for live attenuated vaccine development, regardless of the virus system being explored, is virus yield. For a successful vaccine candidate, it must be possible to produce that vaccine virus in high amount. However, the described large‐scale recoding strategies, while attenuative and in some cases protective from heterologous virus challenge, result in marked defects in virus production levels. The discovery of ZAP as a CpG sensor could provide a potential route to circumvent this issue. Vaccine candidate viruses can simply be grown in ZAP knockout systems, thus recapitulating wildtype virus titres—assuming other unintended effects of mutagenesis (e.g., on genome replication and/or packaging as discussed above) are avoided.

Let us consider the example of IAV as a candidate virus for which a large scale recoded virus could be developed for vaccination. IAV live attenuated vaccines are most commonly produced in embryonated hen's eggs, although there is a motion to switch production to cell culture‐based systems (Perdue et al., 2011). A CpG enriched virus could be—and has been (Gaunt et al., 2016)—produced. One can therefore envisage synthesis of a CpG enriched IAV that replicates to wildtype levels in a CpG‐sensor knockout system (whether this virus is manufactured in a cell culture system or in embryonated hen's eggs; there is emerging technology for creating gene edited chickens; Long et al., 2019; Idoko‐Akoh et al., 2018). This CpG‐enriched virus may offer enhanced immunogenicity (Gaunt et al., 2016) and so the amount of vaccine virus required per dose might also be reduced.

IAV offers a very attractive vaccine target for which synonymous recoding must be a serious consideration. For IAV live attenuated vaccines, PR8 strain—which is nonpathogenic in humans—is used as the backbone, and it is straightforward to switch in synonymously recoded segments into this backbone using well established reverse genetics systems (Fodor et al., 1999; Neumann et al., 1999). By contrast, if we were to recode viable segments of a recombinant virus such as SARS‐CoV‐2 and use that as a vaccine (Digard et al., 2020), there is the risk that this virus can recombine and revert to virulence even with large scale recoding. For poliovirus, the same caveats apply, as well as concerns that this is a neurotropic virus and a CpG‐enriched virus may not be subject to the same replicative losses in the immunoprivileged replication sites (Gao et al., 2015).

It is critical that we fully understand the mechanism of attenuation imparted by synonymous recoding before we apply this technology to vaccine development. For example, we can ask whether the addition of CpGs really allowed greater visibility of the virus to the innate immune system because of those additional CpG motifs, or has some unpredicted defect in replication been introduced—such as disruption or creation of an alternative open reading frame, packaging signals, splice junction, etc.? A candidate vaccine virus may be a lot closer to reversion than it appears in phenotyping as all these examples could potentially be overcome by a single reversion mutation. If this happened when a dinucleotide modified strain were used as a live attenuated vaccine this could create a virus adept at replication in humans, but which is highly immunogenic and therefore pathogenic.

## 6 CONCLUSION

Three drivers shape the genome composition of viruses—translation, mutation and selection. These result in four types of bias—nucleotide, codon, dinucleotide and codon pair. Systematic recoding of viral genomes to disrupt these frequencies almost universally leads to virus attenuation. Synonymous recoding offers a highly attractive vaccine development strategy with the potential to overcome the yield issues currently thwarting current live attenuated vaccine production efforts. CpG dinucleotide deoptimization alone has available a rescue system in which a vaccine virus could be amplified to wildtype virus titres. No such system exists (yet) for any other deoptimization strategy. However, further work must be undertaken to fully understand the mechanisms impacted by this recoding before we can consider using this approach commercially.

## RESEARCH RESOURCES

Figures 2, 4, 5 and 6 were created using BioRender. We are thankful to Professor Peter Simmonds for provision of SSE software used to analyze the data represented in Figures 1, 3, and 7.

## CONFLICT OF INTEREST

The authors have declared no conflicts of interest for this article.

## AUTHOR CONTRIBUTIONS

**Eleanor Gaunt:** Conceptualization; data curation; formal analysis; writing‐original draft; writing‐review & editing. **Paul Digard:** Conceptualization; writing‐original draft; writing‐review & editing.

## RELATED WIREs ARTICLE

lncRNAs regulate the innate immune response to viral infection

## Data Availability

Data sharing is not applicable to this article as no new data were created or analyzed in this study.
